# Sister haplotypes and recombination disequilibrium: a new approach to identify associations of haplotypes with complex diseases

**DOI:** 10.3389/fgene.2023.1295327

**Published:** 2024-01-16

**Authors:** Shun-Yao Liao, Yuan-De Tan

**Affiliations:** ^1^ Institute of Gerontology, Center for Genetics, Sichuan Academy & Sichuan Provincial People Hospital, University of Electronic Science and Technology of China, Chendu, Sichuan, China; ^2^Inflammatory Bowel and Immunobiology Research Institute, Cedars-Sinai Medical Center, Los Angeles, CA, United States

**Keywords:** sister haplotypes, complex disease, association, recombination interference, Alzheimer disease, linkage disequilibrium, SNPs, coronary artery disease

## Abstract

Haplotype-based association analysis has several advantages over single-SNP association analysis. However, to date all haplotype-disease associations have not excluded recombination interference among multiple loci and hence some results might be confounded by recombination interference. Association of sister haplotypes with a complex disease, based on recombination disequilibrium (RD) was presented. Sister haplotypes can be determined by translating notation of DNA base haplotypes to notation of genetic genotypes. Sister haplotypes provide haplotype pairs available for haplotype-disease association analysis. After performing RD tests in control and case cohorts, a two-by-two contingency table can be constructed using sister haplotype pair and case-control pair. With this standard two-by-two table, one can perform classical Chi-square test to find statistical haplotype-disease association. Applying this method to a haplotype dataset of Alzheimer disease (AD), association of sister haplotypes containing ApoE3/4 with risk for AD was identified under no RD. Haplotypes within gene IL-13 were not associated with risk for breast cancer in the case of no RD and no association of haplotypes in gene IL-17A with risk for coronary artery disease were detected without RD. The previously reported associations of haplotypes within these genes with risk for these diseases might be due to strong RD and/or inappropriate haplotype pairs.

## Introduction

High-throughput sequence technologies enable us to easily genotype dozens of single nucleotide polymorphisms (SNPs) within any interesting gene. Such genome-wide SNP data are rapidly growing in disease association studies (Neale and Sham, 2004; Cheng et al., 2005). The association analysis includes single-SNP(Cordell and Clayton, 2002) and haplotype-based disease associations (Zhao et al., 2003a; Zhao et al., 2003b; Clark, 2004; Niu, 2004). Haplotype-based association analysis has several advantages over single-SNP association analysis (Clark, 2004; Yang et al., 2008). The theoretical evidence is that haplotype-based tests would be more powerful because single-marker linkage-disequilibrium (LD)-based methods may not capture all of the available LD information, which is contained in multi-locus haplotypes (Akey et al., 2001; Schaid, 2006; Wen and Tsai, 2014). Therefore, there have been a lot of reporters of haplotype-disease association studies in recent years. However, to date all associations between haplotypes and complex diseases have not excluded recombination interference among multiple loci within haplotypes and hence some results might be confounded by recombination interference. In addition, although many methods (Akey et al., 2001; Sham et al., 2004; Allen and Satten, 2005, 2007, 2009; Fardo et al., 2011; Wen and Tsai, 2014) can be used to test for haplotype-based association, inappropriate haplotype pairs have broadly been used and might lead to finding spurious haplotype-disease associations. To exclude confounding of recombination interference in haplotype-disease association studies, we here introduce recombination disequilibrium (RD) (Tan, 2020). By following definitions of Hardy-Weinberg disequilibrium (HWD) at one locus and linkage disequilibrium (LD) between two loci (Robbins, 1918; Geiringer, 1944; Lewontin and Kojiana, 1960; Lewontin, 1964; Hill and Robertson, 1968), recombination disequilibrium (RD) is defined among three or more loci (Tan, 2020). Although LD has been widely used in haplotype-disease association studies, LD among multiple loci becomes very complicated and poorly understood due to recombination interference. Hastings (1984) indicated that commonly used measures of linkage disequilibrium are not appropriate for a multilocus system. Thomson and Baur (1984) also showed by an example that combinations of allele frequencies and pairwise linkage disequilibrium terms, which are permissible at two-locus level, may not be permissible at three-locus level. LD between two loci is not important for haplotype association, while recombination interference is a key factor in haplotype analysis because it determines frequencies of haplotypes (gametes) in populations. For example, double crossover types in positive interference status are less than those in independent status. The interference intensity is dependent of distance between two adjacent intervals. In classical genetics, coefficient of coincidence is used to measure crossover interference because of the fact that only positive interference has been discovered. With a great advance of technologies in molecular genetics, in particular, with a broad application of genotyping at molecular markers such as SNPs, negative interference has been observed in all species. Likewise, negative interference intensity becomes stronger as distance between adjacent intervals becomes shorter. Coefficient of coincidence is not available to describe negative interference because it is significantly asymmetric in positive and negative directions. This asymmetry leads to difficulty in testing for positive or negative interference in statistics. However, RD can easily measure positive and negative interferences and can easily be tested by Chi-square test (Tan, 2020). In single locus-disease association, Hardy-Weinberg equilibrium (HWE) test is required because frequencies of gene and genotypes follow HWE, then locus-disease associations found are true. In genome-wide study (GWS), linkage disequilibrium (LD) would result in false locus-disease associations due to the fact that linking of non-risk loci to disease gene alters genotype frequencies. Frequencies of haplotypes in recombination disequilibrium status contain linkage or recombination interference effect and hence would generate false haplotype-disease associations. Therefore, RD test is required in haplotype-based association of diseases. In addition, haplotype pairs are also a very important factor impacting association of haplotypes with diseases because correct factor pair is a necessary condition testing for association between two factors. In this paper, we offered a new approach to study haplotype-disease association. The new approach is based on RD and sister haplotypes. We used four public haplotype-based control-case data to show power and robustness of this method.

## Materials and methods

### Data collection

In our current study, we recruirated four public haplotype datasets: 1) SNP haplotype dataset of Alzheimer disease (AD) consists of 210 cases and 159 non-demented elderly controls downloaded from (Fallin et al., 2001). This haplotype data have 8 SNPs (C19M1∼C19M8) in a 205kbp region that contains ApoE gene on chromosome 19 and constructed two configures: M1M3M4*M6 constructs configure1 and M1M2M5M62 constructs configure 2 where M4* is C19M4 that is part of ApoE-ε4 that is a risk gene increasing risk for AD. 2) Breast cancer haplotype data derived from 560 cases and 354 controls (Faghih et al., 2009) are composed of 8 haplotypes containing three variants (−1512 A/C, −1055 C/T and 2044 G/A) in gene IL-13. 3) haplotypes in interleukin-17A gene with risk for premature coronary artery disease (CAD) composed of four SNPs (rs8193036, rs3819024, rs2275913 and rs8193037) were genotyped in 900 premature CAD patients and 935 health persons (Vargas-Alarcon et al., 2015). 4) COMT haplotype dataset published by Peterson et al. (2010). This dataset has 15 haplotypes consisting of 6 SNPs SNP1(rs1544325), SNP2(rs174674), SNP3(rs7290221) SNP4 (rs2239393), SNP5 (rs4680) in exon4 and SNP6 (rs46462316) in Catechol-O-methyl transferase (COMT) genes.

### Haplotype data quality

Recently many large-scale GWAS analyses have been carried out in samples of several thousands of patients and normal individuals. Large SNP data make it possible to conduct large-scale haplotype association analysis of diseases. One can use the above haplotype estimation methods and software packages to create haplotype data from the SNP data. But before performance of our method for haplotype-disease association analysis, haplotype data are necessarily checked in following aspects: 1) since our method is based on biallelic haplotypes, SNPs with multiple alleles must be removed from haplotypes; 2) data with less than 7 types of haplotypes are not available for RD test; 3) haplotypes consisting of more than 3 SNPs should be dissected into three-SNP haplotypes.

### Construction of sister haplotypes

An important step for finding association of haplotypes with a complex disease of study is to construct sister haplotypes. Since haplotypes consist of four base types in DNA sequence, unlike gametes in classical genetics, it is difficult to determine which haplotypes are paired to be sister haplotypes. To construct sister haplotypes, one is first required to translate notation of DNA base haplotypes into notation of classical genetic genotypes. For doing so, we set three pairs of capital and lower letters, for example, Aa at site1, Bb at site 2 and Cc at site 3. A capital letter is assigned to an allele at one site and a lower letter to another allele. For example, in Table 1, M1M4*M6 has sites1 and 2 with alleles C and T, and site 3 with alleles A and G. But for the convenience of understanding, the best assignment way is that the capital letters are assigned to alleles of parental haplotypes and lower letter is assigned to mutation alleles. The parental type has the largest frequencies. In our current example, the parental haplotype is TTA, so we set *T* = T and *t* = C at site 1, *B* = T and *b* = C at site 2 and *A* = A and *a* = G at site 3. Thus, we can translate 8 DNA haplotypes to 8 genotypes of dominant gametes and determine sister gametes.

**TABLE 1:** Data of haplotypes consisting of three SNPs derived from four-SNP haplotypes in configure 1 (M1M3M4*M6) where M4* is C19M4 that is part of ApoE-ε4.

Hap	Gamete	Freq	Overall	Case	Control	Hap	Gamete	Freq	Overall	Case	Control
	M1M3M4*						M1M4*M6				
TCC	aBc	p4′	0.009	0.013	0	CCA	abC	p2′	0.044	0.063	0
CCC	ABc	P2	0.015	0.023	0	CCG	abc	p1′	0.081	0.101	0.042
TTC	abc	p1′	0.097	0.11	0.063	CTA	aBC	p3	0.202	0.173	0.221
CTC	Abc	p3	0.11	0.141	0.042	CTG	aBc	p4′	0.19	0.129	0.24
TCT	aBC	p3′	0.362	0.285	0.417	TCA	AbC	p4	0.016	0.019	0.007
CCT	ABC	p1	0.378	0.288	0.447	TCG	Abc	p3′	0.09	0.105	0.056
TTT	abC	p2′	0.017	0.124	0.017	TTA	ABC	p1	0.224	0.175	0.258
CTT	AbC	p4	0.014	0.014	0.014	TTG	ABc	p2	0.155	0.235	0.176
	M3M4*M6						M1M3M6				
CCA	AbC	p4	0.02	0.03	0	CCA	aBC	p3	0.211	0.211	0.219
CCG	Abc	p3′	0.004	0.006	0	CCG	aBc	p4′	0.182	0.139	0.228
CTA	ABC	P1	0.422	0.343	0.477	CTA	abC	p2′	0.035	0.055	0.002
CTG	ABc	p2	0.318	0.23	0.387	CTG	abc	p1′	0.089	0.12	0.054
TCA	abC	p2′	0.04	0.052	0.007	TCA	ABC	p1	0.231	0.209	0.258
TCG	abc	P1′	0.167	0.2	0.098	TCG	ABc	p2	0.14	0.127	0.159
TTA	aBC	p3	0.004	0.005	0.002	TTA	AbC	p4	0.009	0.009	0.007
TTG	aBc	p4′	0.027	0.133	0.029	TTG	Abc	p3′	0.105	0.255	0.073

### Construction of two-by-two contingency tables

After sister haplotypes are constructed by using the above method, two-by-two tables are required to be constructed. As an example, two-by-two contingency tables (Table 2) with sister-haplotypes in rows and case-control of AD in columns were made by using data in Table 1.

**TABLE 2:** Four two-by-two tables made by using sister haplotypes and case-control.

	Control	Case		Control	Case		Control	Case		Control	Case
TBA	36	4	tbA	25	0	tBA	32	3	TbA	3	47
tba	13	51	TBa	7	47	Tab	14	51	tBa	30	7

a Haplotype data from Fallin et al., (2009).

### Chi-square test for association between haplotypes and diseases

A pair of sister haplotypes is similar to a pair of alleles at a locus, therefore, a two-by-two contingency table constructed with sister-haplotypes and case-control of a disease satisfies Chi-square test for independence between two variables. Using contingency tables, a null hypothesis that a pair of sister haplotypes is not associated with a disease of study can be tested by using Chi-square with degree of freedom = 1. For haplotypes constructed with three SNPs, we have four pairs of sister haplotypes and hence four null hypotheses that are tested by using Chi-squares. To exclude false associations due to recombination interference, testing for RD in haplotypes in control and case cohorts (Tan, 2020) are required. The method for testing for RD can be found in (Tan, 2020). RD is recombination disequilibrium among multiple loci. Similarly to linkage disequilibrium (LD), strong RD also results in spurious findings in haplotype-disease associations because strong RD would significantly change frequencies of haplotypes: $D_{r}=4\left(p_{1}p_{4}-p_{2}p_{3}\right)$ where $p_{1}=p\left(ABC\right)$ , $p_{2}=p\left(Abc\right)$ , $p_{3}=p\left(ABc\right)$ , and $p_{4}=p\left(AbC\right)$ . The $p_{1}$ is frequency of parental types. The $p_{4}$ is frequency of double crossover, and the $p_{2}$ and $p_{3}$ are frequencies of two single-crossovers. $D_{r}$ reflects difference between frequencies of double-crossover and single-crossovers. The frequency of double-crossover measures linkage intensity of three loci on a chromosome. Strong positive or negative interference would significantly change frequency of double crossover. From $D_{r}$ , we can infer if these loci in haplotypes are strongly linked. Therefore, test for $D_{r}$ can exclude spurious association between haplotypes and disease due to linkage. A diagram for construction of sister haplotype pairs, converting haplotypes to genotype of three loci, RD test, and Chi-square test for association between sister haplotype pairs and a disease of study including a practical example is given in Supplementary Material.

### R package SHAD

R package SHAD (sister haplotype-based association of disease) was designed to implement RD tests and association analysis of haplotype with disease in case and control populations. SHAD package works in R environment and has two functions for haplotype association analysis: One is applied to three-SNP haplotypes and another is applied to m-SNP haplotypes where m>3. Function hapAnalysis is used to analyze three-haplotype association with disease. Three-SNP haplotypes have four pairs of sister haplotypes. It outputs RD, Chi-square results and *p*-value for RD and OR, Chi-square test, and *p*-values for OR in case-control. Function hapADA is used to dissect m-SNP haplotypes into n combinations of three-SNP haplotypes and perform association analysis of sister haplotype pairs with disease in all combinations. SHAD package is available for request.

## Results

In nature populations, sister-gametes may have different frequencies due to mutation, deletion, gene conversion and selection. But the disequilibrium between sister-gametes interestingly allows us to develop a statistical approach to test for association of sister-gametes with a complex disease of study. Under the null RD, if difference between sister-gametes in a patient (case) population is significantly different from the health (control) population, then the sister-gamete disequilibrium would be associated with the disease. Current SNP data provide us with a broad way to study haplotype-disease association. Fallin et al. (2001) reported a SNP haplotype dataset of 210 Alzheimer disease (AD) cases and 159 non-demented elderly controls. They used an EM algorithm to estimate frequencies of haplotype consisting of 8 SNPs (C19M1∼C19M8) in a 205kbp region that contains ApoE gene in chromosome 19. Since they just reported haplotype data of configures 1 and 2 (configure1: M1M3M4*M6 and configure 2: M1M2M5M6) where M4* is C19M4 that is part of ApoE-ε4 that has been found to be a risk gene increasing risk for AD (Corder et al., 1993; Saunders et al., 1993; Strittmatter et al., 1993; Farrer et al., 1997), we here did not consider the other configures. We used the haplotype data of these two configures to test for RD among SNPs and associations between haplotypes and risk for AD. We constructed four combinations of three-locus haplotypes from configure 1 by collapsing the same haplotypes and generated three-locus haplotype data (Table 1). According to Fallin et al. (2001), SNPs C19M1,C19M2, C19M5, and C19M6 followed HWE. No LD occurred between C19M1 and C19M4, between C19M1 and C19M5, between C19M1 and C19M6, between C19M2 and C19M3, between C19M2 and C19M5, and between C19M2 and C19M6, but LD existed between C19M4 and C19M6, between C19M3 and C19M4, between C19M3 and C19M5 and between C19M3 and C19M6. The loci C19M1 and C19M8 flank physical interval of 205 kbp on chromosome 19. Our RD analysis shows that there is no RD among loci C19M1, C19M4, and C19M6, among loci C19M1, C19M3, and C19M6 in the case, control, and overall populations, while loci C19M3, C19M4, and C19M6 had very significant RD in all these three populations (*p* = 0.0014 in overall, *p* = 8.8E-06 in the case population and *p* = 0.044 in the control population, Table 3), which is very consistent with significant LDs between them given by Fallin et al. (2001). In haplotype M1M3M4* combination, we detected RD only in the case population (*p* = 0.0076, Table 3). This may be attributed to strong linkage between C19M3 and C19M4. From two-by-two data, we calculated odds ratios and their Chi-square statistics (Table 4). In haplotype combination of three-SNP M1M3M4*, sister haplotypes CCT and TTC (ABC and abc) and sisterhaplotypes CTC and TCT (Abc and aBC) were associated with risk for AD (*p* <0.05). In haplotype combination of three-SNP M1M4*M6, sister-haplotypes TTA and CCG (ABC and abc) and sister haplotypes TTG and CCA (ABc and abC) were detected to be associated with risk for AD (*p* <0.05). These two three-SNP combinations all contain AD risk factor ApoE-ε4 and had no recombination interference among the three loci. But three-SNP M1M3M6 haplotype combination does not contain AD risk factor ApoE-ε4 (M4), its sister haplotypes TCA and CTG (ABC and abc) were also associated with risk for AD (*p* < 0.05) without RD confounding. Sister haplotypes TCG and CTA (ABc and abC) and sister haplotypes CCA and TTG (aBC and Abc) were very significantly associated with risk for AD (*p* <0.01). This result demonstrates that M3 is also a risk factor of AD (called ApoE-ε3) because in configure 2 (M1M2M5M6) without M3 and M4, none of sister haplotype pairs was found to be significantly associated with risk for AD and no RD among triplet SNPs in all four haplotype combinations (Supplementary Tables S1–S3). As M3, M4 and M6 are tightly linked, associations of the sister haplotypes CTA and TCG (ABC and abc) and sister haplotypes CTG and TCA **(**ABc and abC) with risk for AD in three-SNP M3M4*M6 haplotype combination (*p* < 0.01) were confounded by RD.

**TABLE 3:** RD and chi-square testing RD among three SNPs in four haplotypes (M1M3M4*M6) where M4* is C19M4 that is part of ApoE-ε4.

	Overall	Case	Control	Overall	Case	Control
	M1M3M4*			M1M4*M6		
P1	0.475	0.398	0.51	0.305	0.276	0.3
P2	0.032	0.148	0.017	0.199	0.297	0.176
P3	0.472	0.427	0.459	0.292	0.278	0.277
P4	0.023	0.028	0.014	0.206	0.146	0.247
RD	−0.0042	−0.052	−0.0007	0.0047	−0.042	0.0253
X^2^	0.237	7.135	0.0048	0.041	1.959	0.461
*p*-value	0.626	0.0076	0.944	0.839	0.162	0.497
	M3M4*M6			M1M3M6		
P1	0.589	0.5431	0.575	0.32	0.329	0.312
P2	0.358	0.2818	0.394	0.175	0.182	0.161
P3	0.008	0.0116	0.002	0.316	0.466	0.292
P4	0.047	0.1636	0.029	0.191	0.148	0.235
RD	0.0248	0.086	0.0159	0.0058	-0.036	0.0263
X^2^	10.247	19.72	4.042	0.067	1.488	0.527
*p*-value	0.0014	8.8E-06	0.044	0.795	0.222	0.467

**TABLE 4:** Chi-square test of associations between sister haplotypes and Alzheimer disease.

Sister gametes	OR	Z-value	*p*-value	X^2^	*p*-value	OR	Z-value	*p*-value	X^2^	*p*-value
	M1M3M4*					M1M4*M6				
ABC/abc	0.3674	2.399	0.0165	5.1034	0.0239	0.3008	2.442	0.0146	5.2545	0.0218
ABc/abC	NA	NA	NA	0	1	0	NA	NA	5.2704	0.0216
Abc/aBC	4.7143	3.4	0.0007	11.633	0.0006	0.4208	1.876	0.0607	2.8333	0.0923
AbC/aBc	NA	NA	NA	0.1778	0.6733	5.629	1.508	0.1316	1.443	0.2297
	M3M4*M6					M1M3M6				
ABC/abc	0.3609	3.027	0.0025	8.5851	0.0034	0.3863	2.136	0.0327	3.8709	0.0491
ABc/abC	0.0704	2.499	0.0125	8.164	0.0043	0	NA	NA	7.5555	0.0059
Abc/aBC	NA	NA	NA	NA	NA	0.2794	3.259	0.0011	10.04	0.0015
AbC/aBc	NA	NA	NA	0.1277	0.7208	2.4828	0.728	0.4669	0.0246	0.8753

M4* is C19M4 that is part of ApoE-ε4.

Another haplotype data published by Faghih et al. (2009) provide an opposite example. By using differential analysis method (Faghih et al., 2009), found that two haplotypes (ACA and CCA) of three variants in gene IL-13 were significantly associated with risk for breast cancer. By using our method, we got four pairs of sister haplotypes and their frequencies in the case and control populations (Table 5). But as we predicted, our RD analysis showed that RD>0.02 was extremely significant (*p* = 2.81e-06, 5.12e-05, and 1.53e-07 in overall, control, and case populations, respectively, Table 6). Obviously these three variants are in a very short interval of 3.5kbp (457bp + 3099bp) such that extremely strong negative recombination interference occurred. But interestingly none of sister-haplotype pairs was found to be associated with risk for breast cancer (Table 7). The significant differences in frequencies of haplotypes ACA and CCA between the case and control groups in Faghih et al. (2009) just were due to RD and/or inappropriate haplotype pairs used. We did not find any other reports that variants in gene IL-13 are associated with risk for breast cancer. Another similar example can be found in Vargas-Alarcon et al.’s report of association of haplotypes in interleukin-17A gene with risk for premature coronary artery disease (CAD). Four SNPs (rs8193036, rs3819024, rs2275913 and rs8193037) in gene IL-17A were genotyped in 900 premature CAD patients and 935 health persons (Vargas-Alarcon et al., 2015) performed haplotype-based association analysis of premature CAD using individual and common haplotype pairs (called individual-common haplotype pairs). The common haplotype is TAGG. They found that TAGA was associated with risk for CAD at significance level of *p* <0.05. But TAGA has different alleles at only one locus from the common haplotype TAGG. This association, which is equivalent to SNP-disease association, conflicts with the fact that none of SNPs within gene IL-17A was associated with CAD. Our haplotype analysis indicates that these four SNPs should construct 16 haplotypes, of which only 10 haplotypes were observed with hapview, hence only rs8193036, rs3819024, and rs2275913 are valid to construct 8 haplotypes (see Supplementary Material). The RD test shows that in the premature CAD and control populations a very strong negative recombination interference occurred among these three SNP loci within gene IL17A (Supplementary Material). The RD results ( $D_{r}=$ 0.0199 in the case population with *p* = 7.90E-06 and 0.0274 in the control population with *p* = 1.59E-09) are very agreeable with the fact that these SNPs are in a very short region within gene IL-17A indicated by high LD value (*r* ^2^ >0.9 and D’>0.8). As seen in gene IL-13, none of sister haplotype pairs was found to be associated with risk for CAD (Supplementary Material). This result is well consistent with the result that none of SNPs was found to be associated with risk for CAD (Vargas-Alarcon et al., 2015).

**TABLE 5:** Eight kinds of haplotypes consisting of 3SNP in IL-13 and their distribution in patient and normal populations *^a^*.

Haplotypes	Patient n = 560	Normal n = 354	Sister gametes *^b^*	Frequency
ACA	78 (14%)	69 (20%)	ABg	p_2_
ATA	15 (3%)	4 (1%)	Abg	p_3_'
ACG	302 (54%)	182 (50%)	ABG	p_1_
ATG	29 (5%)	15 (4%)	AbG	p_4_
CCA	7 (1%)	0 (0%)	aBg	p_4_'
CTA	72 (13%)	50 (15%)	abg	p_1_'
CCG	15 (3%)	7 (2%)	aBG	p_3_
CTG	42 (7%)	27 (8%)	abG	p_2_'

^a^ Haplotype data from Faghih et al., 2019.

^b^ site1(locus: -1512 A/C): A=A, C = a; site2(locus: -1055 C/T): C = B, T = b; site 3 (locus: -2044 G/A): A = g and G = G.

**TABLE 6:** RD test for recombination interference among the three loci in gene IL-13.

		Overall	Control	Case
P1	=p_1_+p_1_′	0.67016	0.672464	0.667857
p2	=p_2_+p_2_′	0.246273	0.278261	0.214286
P3	=p_3_+p_3_′	0.042728	0.031884	0.053571
P4	=p_4_+p_4_′	0.053882	0.043478	0.064286
RD		0.02591	0.020365	0.031454
*x* ^2^		21.93546	16.40147	27.54278
P-value		2.81e-06	5.12e-05	1.53e-07

**TABLE 7:** Results for association between sister gametes and risk for breast cancer.

Sister gametes	Odds ratio	*x* ^2^	*p*-value
ABG/ abg	1.15232	2.0896	0.1483
ABg/ abG	0.726708	0.8649	0.3524
aBG/ Abg	0.571428	0.5414	0.4618
AbG/ aBg	-	1.9380	0.1639

To furthermore demonstrate that our method is broadly useful, we constructed an R package SHAD (Supplementary Package and Material) and applied it to a COMT haplotype dataset published by Peterson et al. (2010). This dataset has 15 haplotypes consisting of 6 SNPs in Catechol-O-methyl transferase (COMT) genes. Gene COMT has 6 exons and 5 introns (McGregor, 2014). SNP1(rs1544325), SNP2(rs174674) and SNP3(rs7290221) are located in intron 1 and the intervals between SNPs 1 and 2 and between SNPs 2 and 3 are 2357 bp and 12447bp, respectively. SNP4 (rs2239393) is located in intron 3, SNP5 (rs4680) in exon4 and SNP6 (rs46462316) in intron5. Intervals between SNP2 and SNP4, between SNP4 and SNP5, and between SNP5 and SNP6 are separately 16414bp, 833bp, and 861bp. Since Peterson et al. (2010) did not recognize how to construct sister haplotypes, they used individual-common haplotype pairs in the case and control groups to calculate OR and found that haplotypes GAGAGC and AGCGAC were significantly associated with risk for breast cancer. Our sister haplotype analysis was still based on three-SNP system. Haplotypes consisting of 6 SNPs should have 20 three-SNP haplotype combinations, which are more than 15 haplotypes observed, so many haplotypes were missed. In theory, each three-SNP combination should have 8 haplotypes. In haplotype combination list (Supplementary Table S4), 11 combinations had 6 haplotypes and 8 combinations had 7 haplotypes and only one had 8 haplotypes. Since 6 haplotypes cannot construct valid sister gamete pairs, we removed them from our analysis. For combinations with 7 haplotypes, we assigned frequencies of rare haplotypes in the case and control groups to the missing haplotype in each combination. Thus these 8 combinations each had 8 haplotypes. Using our R package SHAD (Sister-haplotype Association of Disease), we obtained the results of RD and disease association tests. The results summarized in Supplementary Table S5 show that except that combination 19 had no significant RD, the other 7 combinations had very significant RD. Combination 6 (SNP1, SNP3 and SNP5), combination13 (SNP2, SNP3 and SNP6), and combination16 (SNP2, SNP5 and SNP6) had very strong negative recombination interference but in combination 9 (SNP1, SNP4 and SNP6), combination 10 (SNP1, SNP5 and SNP6), combination11(SNP2, SNP 3 and SNP4), and combination12 (SNP2, SNP3 and SNP5) there was very strong positive recombination interference among three SNPs. Unsurprisingly, in all combinations none of sister-haplotype pairs was found to be associated with risk for breast cancer (Supplementary Table S5). These results are completely predicted by recombination interference occurring in so short intervals within the gene and within introns. To our knowledge, COMT is chiefly produced by nerve cells in the brain and its variants were found to be associated with risk for mental illness and schizophrenia, other disorders that affect thought (cognition), emotion, bipolar disorder, panic disorder, anxiety, obsessive-compulsive disorder (OCD), eating disorders, and attention deficit hyperactivity disorder (ADHD) (disease http://ghr.nlm.nih.gov/gene/COMT). So far we have not yet found any other evidence for that variants of COMT are associated with risk for breast cancer.

## Discussion

Theoretically, RD reveals recombination interference among multiple loci in an ideal population because in such a population RD is completely derived from recombination interference. In a natural population, however, in addition to recombination interference, RD may also be derived from selection, mutation, gene conversion, migration and/or genetic drift in a small population because these factors can also alter frequencies of gametes or haplotypes (Tan, 2020). In human local populations, these factors may also result in haplotype-based association of complex diseases. Therefore, RD test is required in haplotype-based association of disease.

Frequencies of haplotypes in natural or human populations can be estimated by using the existing methods such as PHASE (Stephens et al., 2001), fastPHASE (Scheet and Stephens, 2006), BEAGLE (Browning and Browning, 2007), IMPUTE2 (Howie et al., 2009), RCEH (Gao et al., 2009) and MaCH (Li et al., 2010). However, current statistical methods for haplotype-disease association analysis, as seen in the above examples, do not consider recombination interference though LD has been excluded in haplotype-based association analysis of diseases. LD can easily be tested between two loci (Robbins, 1918; Geiringer, 1944; Lewontin and Kojiana, 1960; Lewontin, 1964; Hill and Robertson, 1968) but get very complicated among multiple loci because LD cannot measure recombination interference. Recombination interference becomes strong in a short interval. Recombination interference results in change of frequencies of haplotypes which would lead to spurious association between haplotypes and a complex disease. An example is that association of haplotype in gene IL-17A with CAD reported by Vargas-Alarcon et al. (2015) was due to recombination interference within gene IL-17A. In addition, small populations also result in change of haplotype frequencies because of genetic drift, which leads to false association of haplotypes with the disease. Therefore, in a small population, testing for RD in haplotypes can exclude false hapoltype-disease associations. If no RD in haplotypes is found in control and case populations, identified association of sister haplotypes with a disease of study is acceptable in statistics. For example, M1M3M4* haplotype containing risk factor apoE-ε4 and M1M3M6 haplotype containing risk factor apoE-ε3 were found to be associated with risk for AD in small human population (210 AD cases and 159 non-demented elderly controls) using our sister haplotypes and RD test. ApoE-ε3 (Huang et al., 1995; DeMattos et al., 2001; Hopkins et al., 2002; Sen et al., 2012; Pedachenko et al., 2015; Mahan et al., 2022; Sepulveda-Falla et al., 2022; Mulgrave et al., 2023) and apoE-ε4 (Ayyubova, 2023; Chen et al., 2023; Hamza et al., 2023; Koutsodendris et al., 2023; Pires and Rego, 2023; Sun and Xie, 2023; Zhou et al., 2023) have been verified to be risk factors for AD. Fallin et al. (2001) however found that 3 haplotypes in configure 2 flanking M3 and M4 were significantly associated with risk for AD by using individual-others pairs. However, haplotypes in configure 2 (M1M2M5M6) should not be associated with risk for AD because haplotypes in configure 2 do not contain M3 and M4. For example, three SNPs can construct 8 genotypes ABC, abc, ABc, abC, aBC, Abc, AbC and aBc, if we just consider SNP1 and SNP3 and ignore SNP2, we then have four two-SNP genotypes: AC (ABC and AbC), ac (aBc and abc), Ac (ABc and Abc), aC (aBC and abC) each containing B and b alleles at SNP2 locus. If SNP2 is assumed to be a risk factor, then there should not be associations between SNP1-SNP3 haplotypes and risk for the disease. So (Fallin et al., 2001) findings of haplotypes associated with AD in configure 2 are incorrect.

A null hypothesis for haplotype-disease association is that under recombination equilibrium, if disequilibrium between two sister haplotypes does not result in disease, then difference in frequency between sister haplotypes in the case population should be independent of that in the control population. Since two sister haplotypes, like a pair of alleles at a locus, are respectively derived from father and mother and hence are genetically a pair of sister gametes. It is reasonable to construct two-by-two contingency tables with sister haplotypes and case-control for association test. Therefore, inappropriate haplotype pairs would result in false findings of haplotype-disease associations. For example, in individual-common haplotype pairs (Gaudet et al., 2006; Peterson et al., 2010), only one haplotype (e.g., CTA in Table 5) has different alleles at all three loci from the common haplotype (e.g., ACG in Table 5), while the others have the same alleles at two or one locus with the common haplotype. This means that only one haplotype can be paired with the common haplotype in biology. Individual-others pairs (Fallin et al., 2001), as seen in configure 2, would create an incorrect association between haplotypes and risk for the disease because most of the other haplotypes are irrelevant to this haplotype and cannot be paired with it in biology. In order to validate this conclusion, we applied individual-common haplotype pair and individual-others pair methods to the haplotype data (Table 5) of Faghih et al. (2009) and to a new haplotype dataset (Supplementary Table S6) created by assigning 500 patients to the 8 three-SNP haplotypes using their frequencies in the case population and 400 health individuals to the same 8 haplotypes using their frequencies in the normal population. In the original haplotype data (Table5 or Supplementary Table S6), the individual-common pair and sister haplotype pair methods did not find any association between haplotypes and risk for breast cancer but the individual-other pair method identified that ACA was associated with risk for breast cancer (*p* = 0.03254) (Supplementary Table S7). In the new haplotype data (Supplementary Table S6), both individual-common pair and individual-other pair methods found that haplotypes ACA and ATA were very significantly associated with risk for breast cancer (*p* ≤ 0.005191). The inconsistent results between two datasets with the same haplotype frequencies in the case and control populations indicate that both individual-common pairs and individual-other pairs are incorrect haplotype pairs in association analysis. However, we did not find that four pairs of sister haplotypes were associated with risk for breast cancer (Supplementary Table S7) in the original and new haplotype data, suggesting that sister haplotype pairs are correct pairs for testing for association between haplotypes and risk for disease. These four examples above show that our sister haplotype method based on RD has high-sensitivity and lower specificity. Theoretical analysis show that our method satisfies conditions of independence of two random variables, that is, two sister haplotypes are paired and case and control of disease are also paired. We will use simulation data to show that our method would have higher power, higher ROC courve, and lower FDR in multiple haplotype-disease tests than the other haplortype-based methods in future study.

## Data Availability

The original contributions presented in the study are included in the article/Supplementary Material, further inquiries can be directed to the corresponding author.
