# Supplementary material for: Sister haplotypes and recombination disequilibrium: a new approach to identify associations of haplotypes with complex diseases
Source: Front Genet. 2024 Jan 16;14:1295327. doi: 10.3389/fgene.2023.1295327 (PMC10825010; doi:10.3389/fgene.2023.1295327)
Supplement: Supplementary file 1 [file Table1.DOCX]

**Table S1. Data of three-SNP haplotypes in configure 2**

| Hap | gamete | freq | Overall | Case | Control | Hap | gamete | freq | overall | Case | Control |
| --- | --- | --- | --- | --- | --- | --- | --- | --- | --- | --- | --- |
| M2M5M6 | | | | | | M1M2M5 | | | | | |
| ACA | ABc | p2 | 0.264 | 0.264 | 0.289 | TAC | ABC | p1 | 0.439 | 0.454 | 0.415 |
| ACG | ABC | P1 | 0.298 | 0.298 | 0.287 | CAC | aBC | p2 | 0.123 | 0.099 | 0.161 |
| ATA | Abc | p3’ | 0.026 | 0.026 | 0.027 | TGC | AbC | p4 | 0.028 | 0.003 | 0.066 |
| ATG | AbC | p4 | 0.018 | 0.018 | 0.022 | CGC | abC | p3’ | 0.342 | 0.379 | 0.284 |
| GCA | aBc | p4’ | 0.181 | 0.181 | 0.166 | TAT | Abc | p3 | 0.021 | 0.019 | 0.023 |
| GCG | aBC | p3’ | 0.189 | 0.189 | 0.184 | CAT | aBc | p4’ | 0.023 | 0.023 | 0.026 |
| GTA | abc | P1’ | 0.008 | 0.008 | 0.008 | TGT | Abc | p2’ | 0 | 0 | 0 |
| GTG | abC | p2’ | 0.016 | 0.016 | 0.018 | CGT | abc | p1’ | 0.024 | 0.024 | 0.026 |
| M1M5M6 | | | | | | M1M2M6 | | | | | |
| CCA | abC | p2’ | 0.223 | 0.24 | 0.2 | CAA | aBc | p4’ | 0.062 | 0.059 | 0.073 |
| CCG | abc | p1’ | 0.242 | 0.238 | 0.244 | CAG | aBC | p3 | 0.084 | 0.063 | 0.114 |
| CTA | aBC | p3 | 0.013 | 0.018 | 0.012 | CGA | abc | p1’ | 0.174 | 0.199 | 0.14 |
| CTG | aBc | p4’ | 0.034 | 0.029 | 0.04 | CGG | abC | p2’ | 0.192 | 0.204 | 0.17 |
| TCA | AbC | p4 | 0.222 | 0.195 | 0.254 | TAA | Abc | p2 | 0.228 | 0.211 | 0.243 |
| TCG | Abc | p3’ | 0.245 | 0.262 | 0.227 | TAG | ABC | p1 | 0.232 | 0.262 | 0.195 |
| TTA | ABC | p1 | 0.021 | 0.019 | 0.023 | TGA | AbC | p4 | 0.015 | 0.003 | 0.034 |
| TTG | Abc | p2 | 0 | 0 | 0 | TGG | Abc | p3’ | 0.013 | 0 | 0.032 |
|  |  |  |  |  |  |  |  |  |  |  |  |

Data from D. Fallin, A. Cohen, L. Essioux et al., *Genome Res* **11** (1), 143 (2001)

**Table S2. Frequencies of non-sister haplotypes in overall, case and control populations and RD tests**

|  | overall | case | control | overall | Case | Control |
| --- | --- | --- | --- | --- | --- | --- |
| M2M5M6 | | | | M1M2M5 | | |
| P1 | 0.306 | 0.306 | 0.295 | 0.463 | 0.478 | 0.441 |
| P2 | 0.28 | 0.264 | 0.307 | 0.123 | 0.099 | 0.161 |
| P3 | 0.215 | 0.215 | 0.211 | 0.363 | 0.398 | 0.307 |
| P4 | 0.199 | 0.199 | 0.188 | 0.051 | 0.026 | 0.092 |
| RD | 0.00069 | 0.00413 | -0.0093 | -0.0210 | -0.0269 | -0.0088 |
| X2 | 0.00051 | 0.01841 | 0.0699 | 2.267 | 2.8843 | 0.1236 |
| p-value | 0.9819 | 0.892 | 0.7914 | 0.132 | 0.0894 | 0.725 |
|  | M1M5M6 | | | M1M2M6 | | |
| P1 | 0.263 | 0.257 | 0.267 | 0.406 | 0.461 | 0.335 |
| P2 | 0.223 | 0.24 | 0.2 | 0.42 | 0.415 | 0.413 |
| P3 | 0.258 | 0.28 | 0.239 | 0.097 | 0.063 | 0.146 |
| P4 | 0.256 | 0.224 | 0.294 | 0.077 | 0.062 | 0.107 |
| RD | 0.0098 | -0.0096 | 0.0307 | -0.0095 | 0.0024 | -0.0244 |
| X2 | 0.1437 | 0.0834 | 0.5691 | 0.3833 | 0.0209 | 0.7232 |
| p-value | 0.704 | 0.773 | 0.4506 | 0.535 | 0.885 | 0.395 |

**Table S3. Results of sister haplotype-AD association analysis in four haplotype combinations**

| Sister hap | OR | Z-value | p-value | X^2^ | p-value | OR | Z-value | p-value | X^2^ | p-value |
| --- | --- | --- | --- | --- | --- | --- | --- | --- | --- | --- |
|  | M2M5M6 | | | | | M1M2M5 | | | | |
| ABC/abc | 0.685 | 0.305 | 0.760 | 5.47E-30 | 1 | 1.152 | 0.205 | 0.8379 | 1.82E-31 | 1 |
| ABc/abC | 1.196 | 0.213 | 0.832 | 2.67E-32 | 1 | NA | NA | NA | NaN | NA |
| Abc/aBC | 0.906 | 0.138 | 0.890 | 4.68E-32 | 1 | 1.778 | 0.787 | 0.4314 | 0.1746 | 0.676 |
| AbC/aBc | 0.912 | 0.114 | 0.909 | 5.10E-32 | 1 | 0.08 | 2.029 | 0.0425 | 3.117 | 0.077 |
|  | M1M5M6 | | | |  | M1M2M6 | | | | |
| ABC/abc | 0.78 | 0.336 | 0.737 | 1.60E-30 | 1 | 0.923 | 0.212 | 0.8323 | 0.0015 | 0.968 |
| ABc/abC | NA | NA | NA | NaN | NA | 0.708 | 1.046 | 0.2957 | 0.7804 | 0.377 |
| Abc/aBC | 1.309 | 0.302 | 0.763 | 0.1415 | 0.7068 | 8.027 | 1.37 | 0.1706 | 1.7159 | 0.190 |
| AbC/aBc | 1.025 | 0.04 | 0.968 | 1.05E-30 | 1 | 5 | 1.377 | 0.1686 | 1.0266 | 0.311 |

**Table S4. Combinations of 3 SNPs constructing**

**3-SNP haplotypes in 6-SNP haplotypes**

| combination # | sites in 6-SNP haplotypes | | | number of haplotypes |
| --- | --- | --- | --- | --- |
|  | site1 | site2 | site3 |  |
| 1 | 1 | 2 | 3 | 6 |
| 2 | 1 | 2 | 4 | 6 |
| 3 | 1 | 2 | 5 | 6 |
| 4 | 1 | 2 | 6 | 6 |
| 5 | 1 | 3 | 4 | 6 |
| 6 | 1 | 3 | 5 | 7 |
| 7 | 1 | 3 | 6 | 6 |
| 8 | 1 | 4 | 5 | 6 |
| 9 | 1 | 4 | 6 | 7 |
| 10 | 1 | 5 | 6 | 7 |
| 11 | 2 | 3 | 4 | 7 |
| 12 | 2 | 3 | 5 | 8 |
| 13 | 2 | 3 | 6 | 7 |
| 14 | 2 | 4 | 5 | 6 |
| 15 | 2 | 4 | 6 | 7 |
| 16 | 2 | 5 | 6 | 7 |
| 17 | 3 | 4 | 5 | 6 |
| 18 | 3 | 4 | 6 | 6 |
| 19 | 3 | 5 | 6 | 7 |
| 20 | 4 | 5 | 6 | 5 |

Data from Peterson et al. (2010)

| Table S5. RD among three SNPs and association between sister gametes in 8 haplotypes in COMT genes and risk for cancer | | | | | | | | |
| --- | --- | --- | --- | --- | --- | --- | --- | --- |
| combination 6 | RD test | | |  | Association analysis | | | |
| population | RD | *$\chi^{2}$* | p-value |  | gamete | OR | *$\chi^{2}$* | p-value |
| case | 0.038393 | 61.67858 | 4.00E-15 |  | P1 /P1' | 1.034996 | 2.89E-32 | 1 |
| control | 0.031714 | 37.8256 | 7.74E-10 |  | P2 /P2' | 1.106491 | 6.26E-33 | 1 |
| overall | 0.035035 | 97.18532 | 0 |  | P3 /P3' | 1.344086 | 2.72E-33 | 1 |
|  |  |  |  |  | P4 /P4' | 0.912917 | 2.53E-32 | 1 |
| combination 9 | | | | | | | | |
| population | RD | *$\chi^{2}$* | p-value |  | gamete | OR | *$\chi^{2}$* | p-value |
| case | -0.02240 | 9.406148 | 0.002163 |  | P1 /P1' | 1.033651 | 5.03E-32 | 1 |
| control | -0.02488 | 10.41988 | 0.001247 |  | P2 /P2' | 1.019368 | 1.38E-31 | 1 |
| overall | -0.02358 | 19.72317 | 8.95E-06 |  | P3 /P3' | 0.995304 | 5.24E-33 | 1 |
|  |  |  |  |  | P4 /P4' | 1.700087 | 1.08E-32 | 1 |
| combination 10 |  |  |  |  |  |  |  |  |
| population | RD | *$\chi^{2}$* | p-value |  | gamete | OR | *$\chi^{2}$* | p-value |
| case | -0.02141 | 7.496308 | 0.006183 |  | P1 /P1' | 1.019204 | 1.82E-31 | 1 |
| control | -0.02571 | 10.11992 | 0.001467 |  | P2 /P2' | 0.962773 | 9.23E-32 | 1 |
| overall | -0.02354 | 17.52808 | 2.83E-05 |  | P3 /P3' | 0.945055 | 5.14E-33 | 1 |
|  |  |  |  |  | P4 /P4' | 1.268769 | 5.44E-33 | 1 |
| combination 11 |  |  |  |  |  |  |  |  |
| population | RD | *$\chi^{2}$* | p-value |  | gamete | OR | *$\chi^{2}$* | p-value |
| case | -0.03740 | 21.18099 | 4.18E-06 |  | P1 /P1' | 1.159891 | 1.90E-31 | 1 |
| control | -0.04539 | 30.89103 | 2.73E-08 |  | P2 /P2' | 0.976744 | 9.40E-34 | 1 |
| overall | -0.04144 | 51.73346 | 6.36E-13 |  | P3 /P3' | 0.892178 | 3.11E-31 | 1 |
|  |  |  |  |  | P4 /P4' | 0.925511 | 7.89E-33 | 1 |
| combination 12 |  |  |  |  |  |  |  |  |
| population | RD | *$\chi^{2}$* | p-value |  | gamete | OR | *$\chi^{2}$* | p-value |
| case | -0.05506 | 62.84405 | 2.22E-15 |  | P1_P1' | 1.15984 | 4.83E-33 | 1 |
| control | -0.06141 | 75.84371 | 0 |  | P2_P2' | 0.976744 | 9.40E-34 | 1 |
| overall | -0.05826 | 138.5724 | 0 |  | P3_P3' | 0.892178 | 3.11E-31 | 1 |
|  |  |  |  |  | P4 /P4' | NA | NA | AN |
| combination 13 |  |  |  |  |  |  |  |  |
| population | RD | *$\chi^{2}$* | p-value |  | gamete | OR | *$\chi^{2}$* | p-value |
| case | 0.047170 | 19.85533 | 8.35E-06 |  | P1 /P1' | 1.078722 | 5.05E-33 | 1 |
| control | 0.043877 | 16.06747 | 6.11E-05 |  | P2 /P2' | 0.993113 | 7.34E-33 | 1 |
| overall | 0.045584 | 35.84635 | 2.14E-09 |  | P3 /P3' | 0.810507 | 1.26E-32 | 1 |
|  |  |  |  |  | P4 /P4' | 1.143322 | 2.49E-32 | 1 |
| combination 15 |  |  |  |  |  |  |  |  |
| population | RD | *$\chi^{2}$* | p-value |  | gamete | OR | *$\chi^{2}$* | p-value |
| case | 0.070421 | 59.7185 | 1.10E-14 |  | P1 /P1' | 1.083817 | 9.33E-33 | 1 |
| control | -0.068490 | 74.96371 | 0 |  | P2 /P2' | 1.389474 | 9.42E-33 | 1 |
| overall | 0.069548 | 110.4614 | 0 |  | P3 /P3' | 0.930572 | 3.80E-32 | 1 |
|  |  |  |  |  | P4 /P4' | 1.176895 | 1.59E-33 | 1 |
| combination 16 |  |  |  |  |  |  |  |  |
| population | RD | *$\chi^{2}$* | p-value |  | gamete | OR | *$\chi^{2}$* | p-value |
| case | 0.053733 | 28.43943 | 9.67E-08 |  | P1 /P1' | 1.070527 | 3.61E-31 | 1 |
| control | 0.052333 | 25.5447 | 4.32E-07 |  | P2 /P2' | 0.904949 | 9.39E-33 | 1 |
| overall | 0.053067 | 53.9617 | 2.04E-13 |  | P3 /P3' | 0.863629 | 3.10E-32 | 1 |
|  |  |  |  |  | P4 /P4' | 1.137941 | 8.48E-33 | 1 |
| combination 19 |  |  |  |  |  |  |  |  |
| population | RD | *$\chi^{2}$* | p-value |  | gamete | OR | *$\chi^{2}$* | p-value |
| case | 0.00146 | 0.039111 | 0.843229 |  | P1 /P1' | 0.986367 | 5.47E-33 | 1 |
| control | -0.00667 | 0.809136 | 0.368376 |  | P2 /P2' | 1.009802 | 2.66E-33 | 1 |
| overall | -0.00262 | 0.251094 | 0.616306 |  | P3 /P3' | 0.980243 | 7.53E-33 | 1 |
|  |  |  |  |  | P4 /P4' | 0.96859 | 6.32E-33 | 1 |

Data from Peterson et al (2010)

Table S6. Original and new haplotype datasets

| original haplotype data^a^ | | | | | | |  | new haplotype data^b^ | | | | | |
| --- | --- | --- | --- | --- | --- | --- | --- | --- | --- | --- | --- | --- | --- |
|  | case | |  | control | |  | | | case | |  | control | |
| haplotype | count | freq |  | count | freq | genotype | | | count | freq |  | count | freq |
| ACA | 78 | 0.14 |  | 69 | 0.19 | ABg | | | 140 | 0.14 |  | 152 | 0.19 |
| ATA | 15 | 0.03 |  | 4 | 0.01 | Abg | | | 30 | 0.03 |  | 8 | 0.01 |
| ACG | 302 | 0.54 |  | 182 | 0.51 | ABG | | | 540 | 0.54 |  | 408 | 0.51 |
| ATG | 29 | 0.05 |  | 15 | 0.04 | AbG | | | 50 | 0.05 |  | 32 | 0.04 |
| CCA | 7 | 0.01 |  | 0 | 0.01 | aBg | | | 10 | 0.01 |  | 8 | 0.01 |
| CTA | 72 | 0.13 |  | 50 | 0.14 | abg | | | 130 | 0.13 |  | 112 | 0.14 |
| CCG | 15 | 0.03 |  | 7 | 0.02 | aBG | | | 30 | 0.03 |  | 16 | 0.02 |
| CTG | 42 | 0.07 |  | 27 | 0.08 | abG | | | 70 | 0.07 |  | 64 | 0.08 |
| sum | 560 | 1 |  | 354 | 1 |  | | | 1000 | 1 |  | 800 | 1 |

a: data from Table 5. b: haplotype frequencies in case and control are the same with those in the original data where frequency of CCA was changed from 0 to 0.01 because of balance in the new haplotype dataset.

Table S7. The results of applied three haplotype-dissease association methods to the haplotype data in Table S6.

| individual-common haplotype pairs | | | | | | | | | |
| --- | --- | --- | --- | --- | --- | --- | --- | --- | --- |
| original data | | | |  | |  | new data | | |
| haplotype | case | control |  |  | |  | haplotype | case | control |
| ACG | 302 | 182 |  |  | |  | ACG | 540 | 408 |
| ACA | 78 | 69 |  |  | |  | ACA | 140 | 152 |
| X2= 3.7217, p-value = 0.05371 > 0.05 | | | | | X2 = 7.3286, p-value = 0.006786 < 0.01 | | | | |
| ACG | 302 | 182 |  |  | |  | ACG | 540 | 408 |
| ATA | 15 | 4 |  |  | |  | ATA | 30 | 8 |
| X2 = 1.4974, p-value = 0.2211 > 0.05 | | | | | X2 = 7.2405, p-value = 0.007128 < 0.01 | | | | |
| ACG | 302 | 182 |  |  | |  | ACG | 540 | 408 |
| ATG | 29 | 15 |  |  | |  | ATG | 50 | 32 |
| X2 = 0.08907, p-value = 0.765 > 0.05 | | | | | X2 = 0.49684, p-value = 0.4809 > 0.05 | | | | |
| ACG | 302 | 182 |  |  | |  | ACG | 540 | 408 |
| CCA | 7 | 0 |  |  | |  | CCA | 10 | 8 |
| X2 = 2.7259, p-value = 0.09873 > 0.05 | | | | | X2= 0.014252, p-value = 0.905 > 0.05 | | | | |
| ACG | 302 | 182 |  |  | |  | ACG | 540 | 408 |
| CTA | 72 | 50 |  |  | |  | CTA | 130 | 112 |
| X2 = 0.33902, p-value = 0.5604 > 0.05 | | | | | X2 = 0.82412, p-value = 0.364 > 0.05 | | | | |
| ACG | 302 | 182 |  |  | |  | ACG | 540 | 408 |
| CCG | 15 | 7 |  |  | |  | CCG | 30 | 16 |
| X2 = 0.10451, p-value = 0.7465 > 0.05 | | | | | X2 = 1.2223, df = 1, p-value = 0.2689 > 0.05 | | | | |
| ACG | 302 | 182 |  |  | |  | ACG | 540 | 408 |
| CTG | 42 | 27 |  |  | |  | CTG | 70 | 64 |
| X2 = 0.10451, df = 1, p-value = 0.7465 > 0.05 | | | | | X2 = 1.065, , p-value = 0.3021 > 0.05 | | | | |

| individual-others haplotype pairs | | | | | | | | | |  |
| --- | --- | --- | --- | --- | --- | --- | --- | --- | --- | --- |
| original data | | | |  | |  | new data | | |  |
| haplotype | case | control |  |  | |  | haplotype | case | control | |
| ACA | 78 | 69 |  |  | |  | ACA | 140 | 152 |  |
| others | 482 | 285 |  |  | |  | others | 860 | 648 |  |
| X2 = 4.5696, p-value = 0.03254 <0.05 | | | | | X2= 7.8118, p-value = 0.005191 < 0.01 | | | | |  |
| ATA | 15 | 4 |  |  | |  | ATA | 30 | 8 |  |
| others | 545 | 350 |  |  | |  | others | 970 | 792 |  |
| X2 = 1.8512, p-value = 0.1736> 0.05 | | | | | X2 = 7.6621, p-value = 0.005639 < 0.01 | | | | |  |
| ATG | 29 | 15 |  |  | |  | ATG | 540 | 408 |  |
| others | 531 | 339 |  |  | |  | others | 460 | 392 |  |
| X2 = 0.23911, p-value = 0.6248 > 0.05 | | | | | X2 = 1.4865, p-value = 0.2228 > 0.05 | | | | |  |
| ACG | 302 | 182 |  |  | |  | ACG | 50 | 32 |  |
| others | 258 | 163 |  |  | |  | others | 950 | 768 |  |
| X2 = 0.075939, p-value = 0.7829 > 0.05 | | | | | X2 = 0.80512, p-value = 0.3696 > 0.05 | | | | |  |
| CCA | 7 | 0 |  |  | |  | CCA | 10 | 8 |  |
| others | 553 | 354 |  |  | |  | others | 990 | 792 |  |
| X2= 2.9661, p-value = 0.08503 > 0.05 | | | | | X2 = 0, p-value = 1 . 0.05 | | | | |  |
| CTA | 72 | 50 |  |  | |  | CTA | 130 | 112 |  |
| others | 488 | 304 |  |  | |  | others | 870 | 688 |  |
| X2= 0.20151, p-value = 0.6535 > 0.05 | | | | | X2 = 0.30083, p-value = 0.5834 > 0.05 | | | | |  |
| CCG | 15 | 7 |  |  | |  | CCG | 30 | 16 |  |
| others | 545 | 347 |  |  | |  | others | 970 | 784 |  |
| X2 = 0.20452, p-value = 0.6511 > 0.05 | | | | | X2 = 1.4058, p-value = 0.2358 > 0.05 | | | | |  |
| CTG | 42 | 27 |  |  | |  | CTG | 70 | 64 |  |
| others | 518 | 327 |  |  | |  | others | 930 | 736 |  |
| X2 = 2.7751e-29, p-value = 1 > 0.05 | | | | | X2= 0.084984, p-value = 0.7707 > 0.05 | | | | |  |

| Sister-haplotype pairs | | | | | | | | | |
| --- | --- | --- | --- | --- | --- | --- | --- | --- | --- |
| original data | | | |  |  | new data | | | |
| haplotype | case | control |  |  |  | haplotype | case | control |  |
| p1(ACG) | 302 | 182 |  |  |  | p1(ACG) | 540 | 408 |  |
| p1'(CTA) | 72 | 50 |  |  |  | p1'(CTA) | 130 | 112 |  |
| X2 = 0.33902, p-value = 0.5604 > 0.05 | | | | | | X2 = 0.69758, p-value = 0.4036 > 0.05 | | | |
| p2(ACA) | 78 | 69 |  |  |  | p2(ACA) | 140 | 152 |  |
| p2'(CTG) | 42 | 27 |  |  |  | p2'(CTG) | 70 | 64 |  |
| X2 = 1.415, p-value = 0.2342 > 0.05 | | | | | | X2 = 0.51655, p-value = 0.4723 > 0.05 | | | |
| p3(CCG) | 15 | 7 |  |  |  | p3(CCG) | 30 | 16 |  |
| p3'(ATA) | 15 | 4 |  |  |  | p3'(ATA) | 30 | 8 |  |
| X2 = 0.17841, p-value = 0.6727 > 0.05 | | | | | | X2 = 1.3083, p-value = 0.2527 > 0.05 | | | |
| p4(ATG) | 29 | 15 |  |  |  | p4(ATG) | 50 | 32 |  |
| p4'(CCA) | 7 | 0 |  |  |  | p4'(CCA) | 10 | 8 |  |
| X2 = 1.938, p-value = 0.1639 > 0.05 | | | | | | X2 = 0.025407, p-value = 0.8734 > 0.05 | | | |
