# Supplementary material for: Sister haplotypes and recombination disequilibrium: a new approach to identify associations of haplotypes with complex diseases
Source: Front Genet. 2024 Jan 16;14:1295327. doi: 10.3389/fgene.2023.1295327 (PMC10825010; doi:10.3389/fgene.2023.1295327)
Supplement: Supplementary file 2 [file Table2.DOCX]

**Sister haplotype construction and RD Chi-square test**

| **Haplotype** | **Block** | **Premature CAD** | **Control** | **OR (95% CI)** | **P value** |  |
| --- | --- | --- | --- | --- | --- | --- |
| H1 | *T-A-G-G* | 0.614 | 0.624 | NS | - |  |
| H2 | *T-G-A-G* | 0.095 | 0.105 | NS | - |  |
| H3 | *C-A-G-G* | 0.103 | 0.078 | 1.35 (1.00–1.84) | 0.018 |  |
| H4 | *C-A-G-A* | 0.046 | 0.054 | NS | - |  |
| H5 | *C-G-A-G* | 0.045 | 0.052 | NS | - |  |
| H6 | *T-A-G-A* | 0.035 | 0.017 | 2.09 (1.16–3.76) | 0.003 |  |
| H7 | *T-G-G-G* | 0.021 | 0.025 | NS | - |  |
| H8 | *C-A-A-G* | 0.017 | 0.022 | NS | - |  |
| H9 | *T-A-A-G* | 0.015 | 0.011 | NS | - |  |
| H10 | *C-G-G-G* | 0.011 | 0.011 | NS | - |  |
|  |  |  |  |  |  |  |
|  |  |  |  |  |  |  |
|  |  |  |  |  |  |  |
|  |  |  |  |  |  |  |
| **Block** |  | **Block** |  | **Block** |  | **Block** |
| T-A-G |  | T-A-G |  | T-G-G |  | A-G-G |
| *T-G-A* |  | T-G-G |  | T-A-G |  | *G-A-G* |
| C-A-G |  | *C-A-G* |  | C-G-G |  | A-G-G |
| C-A-G |  | *C-A-A* |  | *C-G-A* |  | *A-G-A* |
| *C-G-A* |  | C-G-G |  | *C-A-G* |  | *G-A-G* |
| T-A-G |  | *T-A-A* |  | *T-G-A* |  | *A-G-A* |
| *T-G-G* |  | T-G-G |  | T-G-G |  | G-G-G |
| *C-A-A* |  | *C-A-G* |  | *C-A-G* |  | A-A-G |
| *T-A-A* |  | T-A-G |  | T-A-G |  | A-A-G |
| *C-G-G* |  | C-G-G |  | C-G-G |  | G-G-G |
| *SNP4 delete* |  | SNP3 delete |  | SNP2 delete |  | SNP1 delete |
| \|  \| \| --- \| |  |  |  |  |  |  |
|  |  |  |  |  |  |  |
|  |  |  |  |  |  |  |
|  |  |  |  |  |  |  |
|  |  |  |  |  |  |  |
|  |  |  |  |  |  |  |
|  |  |  |  |  |  |  |
| **Haplotype** | **Block** | **Premature CAD** | **Control** | **Premature CAD** | **Control** |  |
| H1 | T-A-G | 0.614 | 0.624 | 552.6 | 594.672 |  |
| H2 | T-G-A | 0.095 | 0.105 | 85.5 | 100.065 |  |
| H3 | C-A-G | 0.103 | 0.078 | 92.7 | 74.334 |  |
| H5 | C-G-A | 0.045 | 0.052 | 40.5 | 49.556 |  |
| H7 | T-G-G | 0.021 | 0.025 | 18.9 | 23.825 |  |
| H8 | C-A-A | 0.017 | 0.022 | 15.3 | 20.966 |  |
| H9 | T-A-A | 0.015 | 0.011 | 13.5 | 10.483 |  |
| H10 | C-G-G | 0.011 | 0.011 | 9.9 | 10.483 |  |
|  |  | \|  \| \| --- \| |  |  |  |  |
|  |  |  |  |  |  |  |
|  |  |  |  |  |  |  |
|  |  |  |  |  |  |  |
| **Block** | **Premature CAD** | **Control** | **gamete** | **type** |  |  |
| T-A-G | 584.1 | 610.9 | TAG | p1 |  |  |
| T-G-A | 85.5 | 100.1 | Tag | p2 |  |  |
| C-A-G | 134.1 | 125.8 | tAG | p2' |  |  |
| C-G-A | 40.5 | 49.6 | cag | p1' |  |  |
| T-G-G | 18.9 | 23.8 | TaG | p4' |  |  |
| C-A-A | 15.3 | 21 | tAg | p4' |  |  |
| T-A-A | 13.5 | 10.5 | TAg | p3 |  |  |
| C-G-G | 9.9 | 10.5 | caG | p3' |  |  |
| sum | 901.8 | 952.2 |  |  |  |  |
|  |  |  |  |  |  |  |
|  |  |  |  |  |  |  |
|  |  |  |  |  |  |  |
|  |  |  |  |  |  |  |
|  |  |  |  |  |  |  |
|  |  |  |  |  |  |  |
|  |  |  |  |  |  |  |
|  |  | cad | control |  |  |  |
|  | p1 | 584.1 | 610.9 |  |  |  |
|  | p1' | 40.5 | 49.6 |  |  |  |
| OR | 1.17 |  |  |  |  |  |
| Chi-square | 0.37232 |  |  |  |  |  |
| p-value | 0.5417 |  |  |  |  |  |
|  |  | cad | control |  |  |  |
|  | p2 | 85.5 | 100.1 |  |  |  |
|  | p2' | 134.1 | 125.8 |  |  |  |
| OR | 0.802 |  |  |  |  |  |
| Chi-square | 1.1127 |  |  |  |  |  |
| p-value | 0.2915 |  |  |  |  |  |
|  |  | cad | control |  |  |  |
|  | p3 | 13.5 | 10.5 |  |  |  |
|  | p3' | 9.9 | 10.5 |  |  |  |
| OR | 1.36 |  |  |  |  |  |
| Chi-square | 0.044911 |  |  |  |  |  |
| p-value | 0.8322 |  |  |  |  |  |
|  |  | cad | control |  |  |  |
|  | p4 | 18.9 | 23.8 |  |  |  |
|  | p4' | 15.3 | 21 |  |  |  |
| OR | 1.09 |  |  |  |  |  |
| Chi-square | 1.34E-30 |  |  |  |  |  |
| p-value | 1 |  |  |  |  |  |
|  | \|  \| \| --- \| |  |  |  |  |  |
|  |  |  |  |  |  |  |
|  |  |  |  |  |  |  |
|  |  |  |  |  |  |  |
|  | case | control | overall |  |  |  |
| P1=p1+p1' | 0.69261477 | 0.693753281 | 0.69319959 |  |  |  |
| P2=p2+p2' | 0.243512974 | 0.237165354 | 0.24025239 |  |  |  |
| P3=p3+p3' | 0.025948104 | 0.022047244 | 0.02394435 |  |  |  |
| P4=p4+p4' | 0.037924152 | 0.047034121 | 0.04260368 |  |  |  |
| sum | 1 | 1 | 1 |  |  |  |
| RD | 0.019948128 | 0.027401233 | 0.02378017 |  |  |  |
| Chi-square | 19.96277312 | 36.42679977 | 55.7069144 |  |  |  |
| p-value | 7.90E-06 | 1.59E-09 | 8.42E-14 |  |  |  |

**R package SHAD for implement RD tests and association analysis of haplotype with disease in case-control population**

## This R package called SHAD has two functions for haplotype association analysis:

**##** One is applied to independent three-SNP haplotypes and another one is applied to m-SNP haplotypes where m>3.

**##** function hapAnalysis is used to analyze three-haplotype association with disease. Three-SNP haplotypes should have four pairs of sister haplotypes. It outputs RD, chi-square statistics and p-value for RD, OR and chi-square test and p-values for OR in case-control.

## function hapADA is used to dissect m-SNP haplotypes into n combinations of three-SNP haplotypes and perform association analysis of sister haplotype pairs with disease in all combinations.

## In the following example, we display how to dissect 6-SNP haplptypes into 20 combinations of three-SNP haplotypes and how to perform function hapAnalysis to analyze association analysis of sister haplotype pairs with disease breast cancer in case-control way.

## set up local computer path in R console like

setwd("E:/Yuande14/RD/")

source(“C:/R_proprams/ HapAnalysis.R”)

source(“C:/R_proprams/ HapAnalysis1.R”)

## input haplotype data. The data have three columns: haplotype, case and control

hapdata<-read.csv("COMT_haplotype_breastCasncer_data.csv")

# retrieve frequencies of haplotypes in case and control

case<-hapdata$Cases

control<-hapdata$Controls

# haplotype consists of 6 SNPs

cn<-6

rn<-length(case)

#using strsplit to split haplotype string into a set of individual letters

hapn<-unlist(strsplit(haplotype,split=""))

# construct a haplotype matrix hapm with rn rows and cn columns

hapm<-matrix(NA,nrow=rn,ncol=cn)

k<-0

for(i in 1:rn){

for(j in 1:cn){

k<-k+1

hapm[i,j]<-hapn[k]

}

}

x1<-hapm[,2]

x2<-hapm[,3]

x3<-hapm[,4]

# set a matrix for haplotype ID, there are 20 combinations of three SNPs

# the last column is for numbers of haplotypes in each combination

hapid<-matrix(NA,20,4)

N=0;

for(i in 1:4){

for(j in (i+1):5){

for(k in (j+1):6){

N<-N+1

hapid[N,1]<-i

hapid[N,2]<-j

hapid[N,3]<-k

hapid[N,4]<-length(unique(paste(hapm[,i],hapm[,j],hapm[,k])))

}

}

}

colnames(hapid)<-c("site1","site2","site3","number")

# number of the rare haplotype is 78 in case and 54 in control

case[16]<-78

control[16]<-74

#display haplotype # in combination 9

hapid

# combination 9 consists of SNPs 1,4 and 6:

x1<-hapm[,1]

x2<-hapm[,4]

x3<-hapm[,6]

#display types of haplotypes and check which one is absent

hap9<-unique(paste(hapm[,1],hapm[,4],hapm[,6]))

t(t(hap9))

#site1:4A,3G; site2: 3A,4G, site3: 4C,3T

# so missed haplotype is GAT

x1[16]<-"G"

x2[16]<-"A"

x3[16]<-"T"

#perform haplotype association and RD analysis using function hapAnalysis

result9<-hapAnalysis(x1,x2,x3,case,control)

# display results

result9

# save results in a csv file

write.csv(result9,file="COMT_Haplotype9_analysis_result.csv",row.names=FALSE,quote =FALSE)

hapid

#combination 10 consists of SNPs 1,5 and 6

x1<-hapm[,1]

x2<-hapm[,5]

x3<-hapm[,6]

#display types of haplotypes and check which one is absent

hap10<-unique(paste(hapm[,1],hapm[,5],hapm[,6]))

t(t(hap10))

#site1:4A,3G; site2: 4A,3G, site3: 4C,3T , so missed haplotype is GGT

x1[16]<-"G"

x2[16]<-"G"

x3[16]<-"T"

result10<-hapAnalysis(x1,x2,x3,case,control)

result10

write.csv(result10,file="COMT_Haplotype10_analysis_result.csv",row.names=FALSE,quote =FALSE)

# display haplotype IDs in combination 11

hapid

combination 11

x1<-hapm[,2]

x2<-hapm[,3]

x3<-hapm[,4]

#display types of haplotypes and check which one is absent

hap11<-unique(paste(hapm[,2],hapm[,3],hapm[,4]))

t(t(hap11))

#site1:3A,4G; site2: 4C,3G, site3: 4A,3G

x1[16]<-"A"

x2[16]<-"G"

x3[16]<-"G"

result11<-hapAnalysis(x1,x2,x3,case,control)

result11

#write.csv(result11,file="COMT_Haplotype11_analysis_result.csv",row.names=FALSE,quote =FALSE)

#hapid

#combination 13

x1<-hapm[,2]

x2<-hapm[,3]

x3<-hapm[,6]

hap13<-unique(paste(hapm[,2],hapm[,3],hapm[,6]))

t(t(hap13))

#site1:3A,4G; site2: 4C,3G, site3: 4C,3T

x1[16]<-"A"

x2[16]<-"G"

x3[16]<-"T"

result13<-hapAnalysis(x1,x2,x3,case,control)

result13

write.csv(result13,file="COMT_Haplotype13_analysis_result.csv",row.names=FALSE,quote =FALSE)

hapid

#combination 15

x1<-hapm[,2]

x2<-hapm[,4]

x3<-hapm[,6]

hap15<-unique(paste(hapm[,2],hapm[,4],hapm[,6]))

t(t(hap15))

#site1:3A,4G; site2: 4G,3A, site3: 4C,3T

x1[16]<-"A"

x2[16]<-"A"

x3[16]<-"T"

result15<-hapAnalysis(x1,x2,x3,case,control)

#result15

write.csv(result15,file="COMT_Haplotype15_analysis_result.csv",row.names=FALSE,quote =FALSE)

hapid

#combination 16

x1<-hapm[,2]

x2<-hapm[,5]

x3<-hapm[,6]

hap16<-unique(paste(hapm[,2],hapm[,5],hapm[,6]))

t(t(hap16))

#site1:3A,4G; site2: 4A,3G, site3: 4C,3T

x1[16]<-"A"

x2[16]<-"G"

x3[16]<-"T"

result16<-hapAnalysis(x1,x2,x3,case,control)

#result16

write.csv(result16,file="COMT_Haplotype16_analysis_result.csv",row.names=FALSE,quote =FALSE)

hapid

x1<-hapm[,3]

x2<-hapm[,5]

x3<-hapm[,6]

hap19<-unique(paste(hapm[,3],hapm[,5],hapm[,6]))

t(t(hap19))

#site1:4G,3C; site2: 4A,3G, site3: 4C,3T

x1[16]<-"C"

x2[16]<-"G"

x3[16]<-"T"

result19<-hapAnalysis(xx1=x1,xx2=2,xx3=x3,case,control)

result19

write.csv(result19,file="COMT_Haplotype19_analysis_result.csv",row.names=FALSE,quote =FALSE)
